# Babies born in the pre-hospital setting attended by ambulance clinicians in the north east of England

**DOI:** 10.29045/14784726.2019.12.4.3.43

**Published:** 2019-12-01

**Authors:** Graham McClelland, Emma Burrow, Helen McAdam

**Affiliations:** North East Ambulance Service NHS Foundation Trust: ORCID iD: http://orcid.org/0000-0002-4502-5821; North East Ambulance Service NHS Foundation Trust; North East Ambulance Service NHS Foundation Trust

**Keywords:** ambulance, childbirth, pre-hospital

## Abstract

**Introduction:**

The majority of births in the United Kingdom happen in hospital or at stand-alone midwife led centres, or with the support of midwives in a planned fashion outside of hospital. The unplanned birth of a baby in the pre-hospital setting is a rare event which may result in an ambulance being called, so attendance at a birth is a rare event for ambulance clinicians. A service evaluation was conducted to report which clinical observations were recorded on babies born in the pre-hospital setting who were attended by ambulance clinicians from the North East Ambulance Service (NEAS) over a one-year period.

**Methods:**

A retrospective service evaluation was conducted using routinely collected data. All electronic patient care records covering a one-year period between 1 October 2017 and 30 September 2018 with a primary impression of ‘childbirth’ were examined.

**Results:**

This evaluation identified 168 individual pre-hospital childbirth cases attended by NEAS clinicians during the evaluation timeframe. The majority (85%) of babies were born to multiparous mothers with a median gestation of 39 weeks. Very few clinical observations were recorded on the babies (respiratory rate 23%, heart rate 21%, temperature 10%, APGAR 8%, blood sugar 1%) and no babies had all five of these observations documented. Only 5% of babies had any complications documented.

**Conclusion:**

This study showed that NEAS ambulance clinicians rarely attend babies born in the pre-hospital setting and that complications were infrequently recorded. There was a lack of observations recorded on the babies, which is an issue due to the clear link between easily measurable characteristics such as temperature and mortality and morbidity.

## Introduction

The majority of births in the United Kingdom occur in hospital or at stand-alone midwife led centres, or with the pre-planned support of midwives outside of hospital. The birth of a baby in the pre-hospital setting is often unplanned and attendance at a birth is an uncommon event for ambulance clinicians. The rate of babies born before arrival (BBA) at hospital in the United Kingdom was reported as 0.5% (Loughney, Collis, & Dastgir, 2006). The rate of babies BBA in Australia (Thornton & Dahlen, 2018) and Ireland (Unterscheider, Ma’ayeh, & Geary, 2011) was similar to the United Kingdom, whereas in the United States the rate was higher at 1.4% (MacDorman, Mathews, & Declercq, 2014).

Babies born in an unplanned fashion in the pre-hospital setting have an increased risk of complications such as hypothermia, perinatal mortality, low birth weight and admission to neonatal intensive care (Loughney et al., 2006; Thornton & Dahlen, 2018; Unterscheider et al., 2011). Despite these increased risks, the literature indicates that most pre-hospital births require minimal interventions from ambulance clinicians (McLelland, McKenna, Morgans, & Smith, 2018).

A recent update to the UK Ambulance Service Clinical Practice Guidelines (Joint Royal Colleges Ambulance Liaison Committee & Association of Ambulance Chief Executives, 2017) revised the guidance for ‘Care of the newborn’ with specific mention of the risks of hypoxia, hypoglycaemia and hypothermia for newborn babies. Based on these guidelines, a minimum basic set of clinical observations that should be recorded on all newborns included pulse rate and respiratory rate, both of which also feature in the APGAR (appearance, pulse rate, grimace, activity, respiration) score (Apgar, 1953), blood sugar (BM) and temperature.

The aim of this service evaluation was to report if these clinical observations were being fully recorded on babies born in the pre-hospital setting who were attended by ambulance clinicians from the North East Ambulance Service (NEAS). This evaluation was conducted as the data describing the pre-hospital care of these patients are scarce, to identify areas for improvement and to support future research.

## Methods

A retrospective service evaluation was conducted using routinely collected data to determine what clinical observations were recorded on babies born in the pre-hospital setting who were attended by NEAS clinicians over a one-year period.

### Setting

NEAS is the regional ambulance provider for around 2.5 million people in north east England, covering Northumberland, Tyne and Wear, County Durham, Darlington and Teesside. NEAS employs around 1200 ambulance clinicians (paramedics and other clinical roles) who work out of 56 stations across the region (North East Ambulance Service NHS Foundation Trust, 2019).

### Data collection, extraction and analysis

All electronic patient care records (ePCRs) covering a one-year period between 1 October 2017 and 30 September 2018 with an ambulance clinician recorded primary impression of ‘childbirth’ were requested from the NEAS informatics team.

The ePCRs were then filtered based on the following inclusion and exclusion criteria:

Inclusion:

- Baby born in the pre-hospital setting

Exclusion:

- Baby born in hospital
- Midwife in attendance at pre-hospital birth
- Miscarriage or still birth

The following data were manually extracted from the ePCRs by the authors:

- Relevant timings (call to hospital, on scene time, birth to hospital)
- Destination hospital
- Age of mother
- Parity (baby number)
- Gestation
- Clinical observations recorded on the baby (temperature, BM, heart rate, respiratory rate, APGAR)
- Complications and interventions performed

These items were selected by the authors as being relevant to care of the newborn, available in the pre-hospital setting and included in national ambulance guidelines (Joint Royal Colleges Ambulance Liaison Committee & Association of Ambulance Chief Executives, 2017).

All data are presented in a descriptive fashion along with the number of cases where data were available. Summary statistics are presented using median, interquartile range (IQR) and range. Complications and interventions were identified and extracted from the narrative and treatment sections of the ePCR and are presented in an aggregate fashion due to the small number of patients involved.

## Results

There were 236 ePCRs with ‘childbirth’ recorded as the impression between 1 October 2017 and 30 September 2018. The ePCRs were filtered using the inclusion and exclusion criteria, which resulted in 168 individual childbirth cases being included in this service evaluation.

From the original 236 ePCRs identified, 68 cases were excluded for the following reasons: baby transported prior to crew’s arrival (n = 1); babies born and cared for by midwives (n = 2); duplicate cases (n = 24); miscarriages (n = 6); not childbirth (n = 5); still birth (n = 1); transported to hospital for birth (n = 29).

### Relevant timings

The median call to hospital time (n = 146) was 65 minutes (IQR 51–86, range 25–194). The median on scene time (n = 149) was 30 minutes (IQR 22–41, range 8–113). The median birth to hospital time (n = 138) was 55 minutes (IQR 39–72, range 10–192).

The times when the calls leading to the pre-hospital birth were received by the ambulance service were examined (n = 154), with 23% of calls in office hours (0900–1700) and 63% overnight (2000–0800).

### Destination hospital

One hundred and sixty-six babies were transported to 10 different hospitals across the north east; two babies were not transported as midwives arrived after the babies were born and stood the ambulance crews down.

### Age of mother

The median age of the mother (n = 166) was 28 years (IQR 24–32, range 15–43).

### Parity

**Figure 1:**
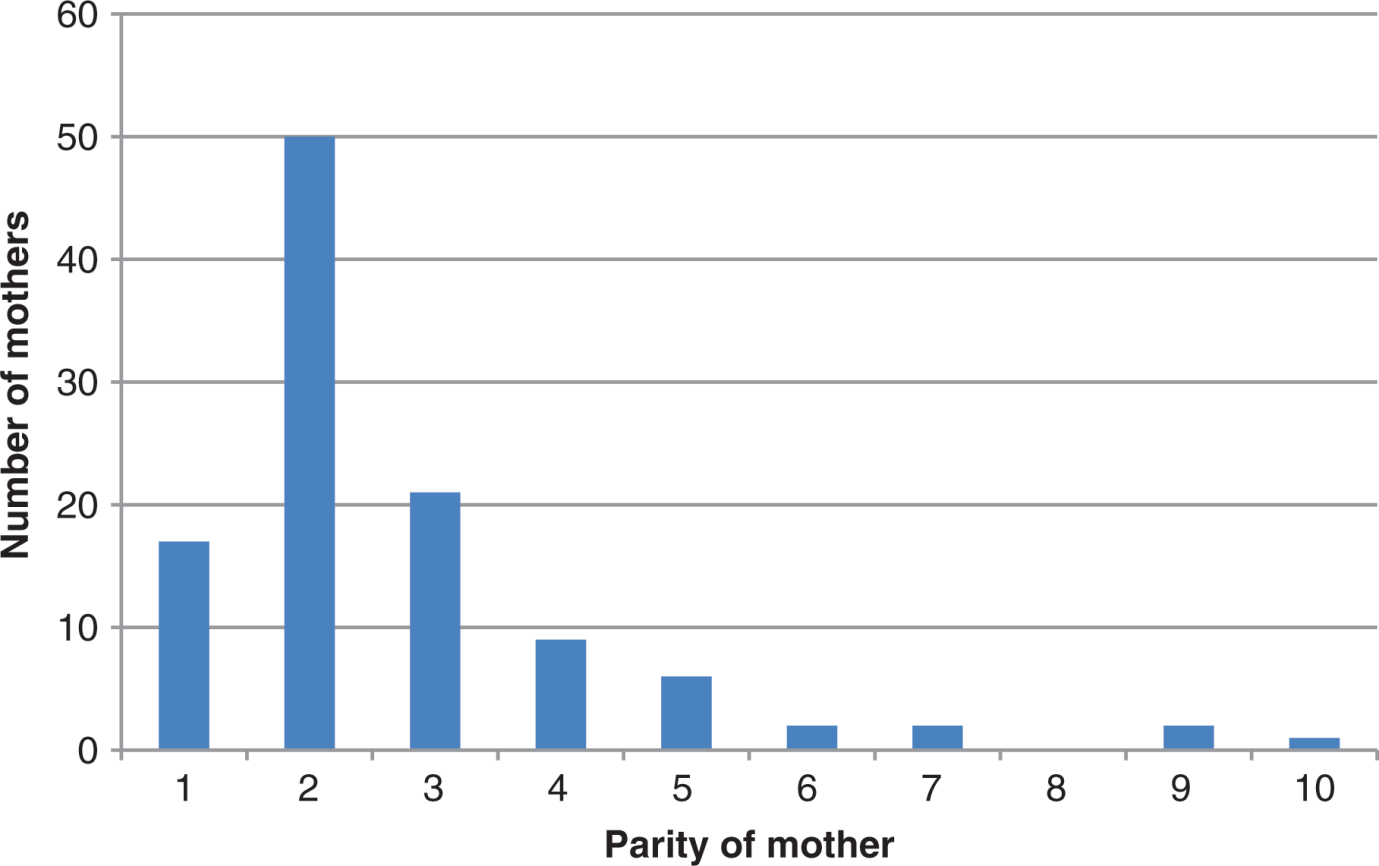
Figure 1. Parity of mothers attended by ambulance clinicians.

Parity was recorded in 110 cases, with the median value of second baby (IQR 2–3, range 1–10; Figure 1).

### Gestation

The median gestation period (n = 78) was 39 weeks (IQR 38–40, range 19–42).

### Clinical observations recorded on the baby

Eighteen (10%) babies had a temperature recorded. In 14 of these cases a single temperature was recorded and in four cases two temperatures were recorded. The median temperature was 36.3°C (IQR 35.5–36.5, range 34.8–37.5). The location where the temperature was taken from was recorded in 13 cases: axillary (n = 2); temporal (n = 2); tympanic (n = 9).

APGAR was recorded for 13 (8%) babies, with one baby scoring eight, one baby scoring nine and 11 babies scoring 10.

Two (1%) babies had a BM recorded (3.1 and 5.9).

Thirty-five (21%) babies had a heart rate recorded. In 28 cases a single heart rate was recorded, in five cases two heart rates were recorded and two cases had four heart rates recorded. The median heart rate was 150 (IQR 136–160, range 100–200).

Thirty-eight (23%) babies had a respiratory rate recorded. In 30 cases a single respiratory rate was recorded, in six cases two respiratory rates were recorded and in two cases four respiratory rates were recorded. The median respiratory rate was 38 (IQR 35–45, range 18–65).

**Figure 2:**
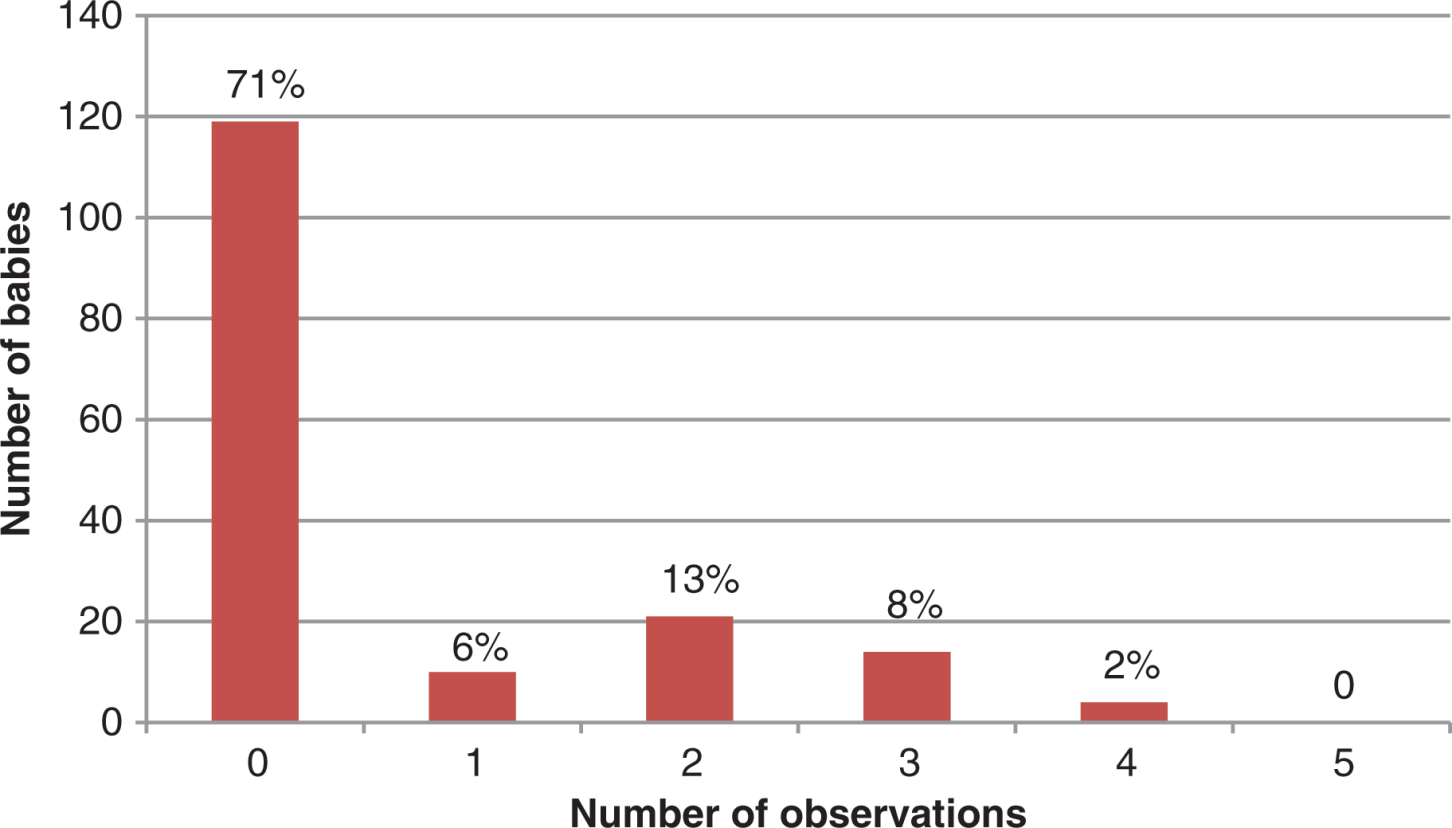
Figure 2. Total number of observations documented on babies.

The total number of observations included in the basic minimum set defined earlier (pulse rate, respiratory rate, APGAR, BM, temperature) documented on each baby is displayed in Figure 2.

### Complications and interventions performed

Ninety-seven (58%) babies had some form of warming recorded involving combinations of blankets, clothing and hats, towels and foil/blizzard blankets.

Nine (5%) cases had complications documented including issues with the umbilical cord and concerns around breathing. Six (4%) cases involved inflation breaths and/or oxygen administration to the baby. Three (2%) cases had CPR or resuscitation documented.

Eight (5%) mothers received syntometrine, with one patient receiving tranexamic acid.

## Discussion

### Description of results

This service evaluation describes the clinical data recorded on babies born in the pre-hospital setting attended by NEAS and shows that very few observations, including those recommended in national guidelines, were documented. No babies had all five basic observations documented and 71% had none of these observations documented. Of pre-hospital births attended by ambulance clinicians, 93% had no complications or interventions recorded, so the omission of basic observations is difficult to explain.

### Results in context

The mothers described in this study were slightly younger than the national average which was reported as 30.5 in 2017 (Office for National Statistics, 2017), but were similar in age to previous research in the north east which reported a mean age of 27.6 (Loughney et al., 2006). Three quarters of calls leading to pre-hospital births occurred out of office hours, which replicates the findings of previous research (Javaudin et al., 2019; McLelland et al., 2018; Unterscheider et al., 2011). The reasons for the increased frequency of pre-hospital births outside of office hours are unclear.

Previous research reporting babies’ BBA (Javaudin et al., 2019; Loughney et al., 2006; McLelland et al., 2018; Unterscheider et al., 2011) described a similar pattern to the data reported here in that multiparous women were more frequent than primiparous women and that babies were born at, or around, full term. Higher parity is known to be associated with more rapid labour (Gunnarsson, Skogvoll, Jonsdottir, Roislien and Smarason, 2017).

There was a low rate of recording of all of the clinical observations reported. Only 10% of babies had a temperature recorded and 72% (n = 13) of these were below 36.5°C, which is the lower limit recommended by the World Health Organization (World Health Organization & Maternal and Newborn Health/Safe Motherhood, 1997) and the International Liaison Committee on Resuscitation (ILCOR) Neonatal Task Force (Perlman et al., 2015). A similar study by Flanagan, Lord and Barnes (2017) conducted in Australia reported that only 2% of babies delivered by paramedics had a temperature recorded and all of these were hypothermic (< 36.2°C).

The maintenance of normothermia (36.5–37.5°C) has been shown to be very important to newborns, and deviations from this range, particularly hypothermia, are linked to increased mortality and morbidity (Chitty & Wyllie, 2013; Lunze, Bloom, Jamison, & Hamer, 2013; Trevisanuto, Testoni, & de Almedia, 2018). Evidence suggests a direct relation between hypothermia and mortality, with every 1°C below 36.5°C increasing the risk of mortality by 28% (Perlman et al., 2015). A recent study by Javaudin et al. (2019) using multi-variate logistic regression identified four factors which were predictive of neonatal mortality and morbidity: multiparity, prematurity, maternal pathology and hypothermia. This is important as hypothermia is the only modifiable condition in the pre-hospital setting.

### Limitations and strengths

This evaluation is limited by the retrospective nature of the data and the potential for observations and interventions to not have been documented. The small number of births could also be a limitation of this study. However, the total number of live births in the north east in 2016 was 28,574 (Office for National Statistics, 2018). Applying a pre-hospital birth rate of 0.5% to this figure and assuming there has been little change between 2016 and 2017/18, the expected rate of pre-hospital births would be 143, so the population of 168 reported in this study appears plausible. The detailed case by case examination of the primary documentation is a strength of this study; however, the variation in how pre-hospital births were recorded meant that the data extraction involved some subjective interpretation.

### Generalisability

There is no reason to believe that pre-hospital births in the north east differ significantly from pre-hospital births across the United Kingdom; therefore, the data from this service evaluation should be generalisable to other areas. However, a national audit or database of pre-hospital births, which would be beneficial for research and development in this area, would be needed to prove or disprove this assertion. NEAS clinicians are trained in a similar fashion and to a similar standard to other UK pre-hospital clinicians, and ambulance protocols across the United Kingdom are based on national guidelines such as JRCALC (Joint Royal Colleges Ambulance Liaison Committee & Association of Ambulance Chief Executives, 2017); however, different ambulance trusts will interpret and implement protocols based on local needs, geography and expertise. In addition, different ambulance services use different formats of patient records, which may introduce variation into which data are recorded on pre-hospital births.

### Controversies

The low rate of recording of basic observations is worrying and the reasons for this need to be explored. Possible explanations for this lack of recorded observations could be: a lack of confidence in taking neonatal observations; thinking taking and/or recording the observations was unnecessary in babies that appear well; lack of equipment (e.g. saturation probes and blood pressure cuffs) designed for neonates; and other reasons.

### Implications for practice

The number of pre-hospital births can be used to estimate the exposure of ambulance clinicians to these events. If 168 births are evenly distributed between approximately 1200 clinicians (North East Ambulance Service NHS Foundation Trust, 2019), then the exposure rate would be 0.14 births per year per clinician. In practice, the majority of clinicians work on double crewed ambulances so the exposure rate could be doubled to 0.28 per year. This represents a very low level of exposure and concerns have been raised around skill maintenance and competency in other situations that clinicians are infrequently exposed to (Dyson et al., 2016).

Ambulance clinician education and training may need to reinforce the reasons why taking, recording and acting on observations such as temperature in a newborn baby are important in order to change practice going forward. In order to enable good practice, equipment suitable for taking observations on newborn babies needs to be available.

### Implications for research

Further research is needed to more comprehensively describe the clinical characteristics of babies born in the pre-hospital setting. These data could potentially be used to explore any links between pre-hospital birth, the initial clinical presentation of the baby, the need for treatment in or out of hospital and longer-term outcomes.

## Conclusion

This study shows that NEAS ambulance clinicians rarely attend babies born in the pre-hospital setting, and when they are present the births are often uncomplicated and require few interventions. There was a lack of observations recorded on the babies which is an issue due to the clear link between easily measurable characteristics such as temperature and mortality and morbidity. More research is needed to establish the barriers and facilitators to taking and recording observations on babies. Consistent recording of these data would allow areas for improvement to be identified and linking the pre-hospital data to hospital data would allow longer-term implications and outcomes to be explored.

## Author contributions

All authors contributed to the study and read and approved the final manuscript.

## Conflict of interest

None declared.

## Ethics

Not required.

## Funding

None.

## Notes

A correction to this article was published in McClelland, G., Burrow, E., & McAdam, H. (2022). Correction to published conflict of interest statement in ‘Babies delivered by ambulance clinicians in the North East of England: a service evaluation’. *British Paramedic Journal*, *7*(2), 58.
