# Correction to published conflict of interest statement in ‘Babies delivered by ambulance clinicians in the North East of England: a service evaluation’

**DOI:** 10.29045/14784726.2022.09.7.2.58

**Published:** 2022-09-01

**Authors:** Graham McClelland

**Affiliations:** Editor of the *BPJ*

In ‘Babies delivered by ambulance clinicians in the North East of England: a service evaluation’ by McClelland, Burrow and McAdam which was published by the *British Paramedic Journal* on pages 43–48 in issue 4(3) in December 2019, there was an error of omission in the conflicts of interest statement on page 47. No conflicts of interest were printed, when the fact that McClelland was a member of the editorial board of the *British Paramedic Journal* at the time of submission should have been declared. This has been discussed by the editorial board and processes have been implemented to reduce the likelihood of this type of error happening again.
